# High-Resolution Black Blood Vessel Wall Imaging in COVID-19 Encephalopathy—Is it Really Endotheliitis?

**DOI:** 10.1007/s00062-021-01109-y

**Published:** 2021-10-28

**Authors:** Konstanze V. Guggenberger, Thorsten A. Bley, Marius L. Vogt, Horst Urbach, Stephan Meckel

**Affiliations:** 1 Department of Diagnostic and Interventional Radiology, University Hospital Würzburg, University of Würzburg, Oberdürrbacher Str. 6, 97080 Wirtzburg, Germany; 2 Department of Diagnostic and Interventional Neuroradiology, University Hospital Würzburg, University of Würzburg, Josef-Schneider-Straße 11, 97080 Wirtzburg, Germany; 3 Department of Neuroradiology, Medical Center, University of Freiburg, Breisacher Straße 64, 79106 Freiburg, Germany; 4 Institute of Neuroradiology, Kepler University Hospital, Johannes Kepler University Linz, Linz, Austria

Dear Editor,

with interest we read the study by Uginet et al. “Cerebrovascular Complications and Vessel Wall Imaging in COVID-19 Encephalopathy—A Pilot Study” [1]. They described the findings on high-resolution black blood vessel wall magnetic resonance imaging (MRI) at 1.5 T that was performed in 34 older patients (66.5 ± 9.2 years; 10.3% female) with coronavirus diseases 2019 (COVID-19) and acute encephalopathy in a retrospective observational study.

The authors reported circumferential vessel wall enhancement in 85% (29/34) patients located in the intracranial vertebral and basilar arteries. The longitudinal extension of this vessel wall enhancement was described between 4.0 mm and 42.0 mm (18.0 ± 9.76 mm) and affected both vertebral arteries in 50% of patients. No associated stenosis of the affected vessels was seen on MR angiography.

The authors concluded that these findings were suggestive of endotheliitis and supportive of an inflammatory origin of COVID-19 encephalopathy; however, they could not demonstrate any association between vessel wall enhancement and ischemic brain lesions, severity of encephalopathy, measured by the confusion assessment method (CAM) scale, or the presence of microbleeds on susceptibility-weighted images. Most importantly, neither histopathological correlation from any of their patients nor a control group of age-matched patients without COVID-19 encephalopathy was presented. Given these limitations, such conclusion cannot be drawn from their descriptive study findings.

To account for their study findings, intracranial vasa vasorum, developing with advancing age, typically causing concentric mural contrast enhancement and vessel wall thickening in vessel wall imaging using black-blood MRI [2], need to be considered. This type of vessel wall enhancement is frequently found on MRI vessel wall images in older subjects along the proximal intracranial vessel segments [3] after the vessels’ dural crossing and may be easily mistaken for inflammatory changes, such as vasculitis. In our recently published study, we analyzed 43 older (mean age 71 years, SD 10 years) subjects without any clinical or laboratory signs of vasculitis and found concentric vessel wall enhancement of the proximal intradural vertebral artery in 39 patients (91%) [4]. Mean longitudinal extension of vessel wall enhancement after dural entry was 13 mm (range 0–52 mm) in the vertebral artery (right vertebral artery mean 12 mm, range 0–34 mm, left vertebral artery 14 mm, range 0–52 mm).

The reported images showing vessel wall enhancement of the proximal vertebral arteries (left vertebral artery in Fig. 1d and Fig 2a–c; bilateral vertebral arteries in Fig. 3d) of the addressed study [1] resemble the appearance of high-resolution vessel wall images from our non-vasculitic cohort (Fig. 2a–c, Fig. 3b in [4]): concentric vessel wall enhancement and slight vessel wall thickening of the non-stenotic vertebral artery in its proximal intradural course. This finding most likely represents vasa vasorum related vessel wall enhancement and should not be misinterpreted as inflammatory affection. Previous histopathologic and imaging studies have revealed results that support the presence of vasa vasorum causing these vessel wall imaging findings [3, 5–7].

We do not intend to refute the results of the addressed study but rather draw attention to the fact that vasa vasorum may constitute a potential confounder in diagnosing endotheliitis or intracranial vasculitis. Vessel wall enhancement in the proximal intradural vessel segments, in particular affecting the proximal vertebral arteries, is a common finding in non-vasculitic older patients without any pathologic significance. Supportive of this interpretation is the fact that COVID-19 related cerebral endotheliitis was hitherto described in small intracerebral vessels in a histopathological study [8].
